# Retraction Note: Piperlongumine inhibits lung tumor growth via inhibition of nuclear factor kappa B signaling pathway

**DOI:** 10.1038/s41598-025-99020-w

**Published:** 2025-04-30

**Authors:** Jie Zheng, Dong Ju Son, Sun Mi Gu, Ju Rang Woo, Young Wan Ham, Hee Pom Lee, Wun Jae Kim, Jae Kyung Jung, Jin Tae Hong

**Affiliations:** 1 College of Pharmacy and Medical Research Center, Chungbuk National University, Osong-eup, Heungduk-gu, Cheongju, Chungbuk 28160 Republic of Korea; 2 New Drug Development Center, KBio, Osong-eup, Heungduk-gu, Cheongju, Chungbuk 28160 Republic of Korea; 3 Department of Chemistry, Utah Valley University, 800 West University Parkway, Orem, UT 84508 USA; 4 College of Medicine, Chungbuk National University, Chungdae-ro 1, Seowon-gu, Cheongju, Chungbuk 28644 Republic of Korea

Retraction of: *Scientific Reports* 10.1038/srep26357, published online 20 May 2016

The Editors have retracted this Article following concerns about image duplication in Figure 7D. The Authors were unable to provide the original images and raw data for verification. The Authors were also unable to provide evidence of ethics approval for this study. The Editors have therefore lost confidence in the veracity of the data presented.

The Authors have not responded to correspondence from the Editors regarding this retraction.

